# Supplementary material for: Bayesian modelling disentangles language versus executive control disruption in stroke
Source: Brain Commun. 2024 Apr 10;6(3):fcae129. doi: 10.1093/braincomms/fcae129 (PMC11069117; doi:10.1093/braincomms/fcae129)
Supplement: fcae129_Supplementary_Data [file fcae129_supplementary_data.docx]

**Supplementary Information**

**Bayesian modeling disentangles language versus executive control disruption in stroke**

Gesa Hartwigsen, Jae-Sung Lim, Hee-Joon Bae, Kyung-Ho Yu, Hugo J. Kuijf, Nick A. Weaver, J. Matthijs Biesbroek, Jakub Kopal, Danilo Bzdok

**Materials and Methods**

**Participant recruitment**

We retrospectively selected 1401 patients from a stroke registry based on Bundang and Hallym Vascular Cognitive Impairment cohorts – prospectively recruited cohorts of patients initially admitted to the Seoul National University Bundang Hospital or Hallym University Sacred Heart Hospital in South Korea between 2007 and 2018^1^. These patients were diagnosed with acute ischaemic infarction based on diffusion-weighted MRI, typically within one week after symptom onset. Among all available patients, a total of 1080 patients were selected based on the following criteria: (1) availability of brain MRI showing acute tissue infarction in the diffusion-weighted imaging (DWI) and/or fluid-attenuated inversion recovery (FLAIR), (2) successful lesion segmentation and registration, (3) no previous cortical infarcts, large subcortical infarcts (>15 mm) or haemorrhages (>10 mm) on MRI, and (4) availability of follow-up data on key demographics and neuropsychological assessment (the 60-min Korean-Vascular Cognitive Impairment Harmonization Standards-Neuropsychology Protocol^2,3^. We excluded patients (1) whose MRI was inadequate for properly obtaining neuroimaging variables, (2) who had a bilateral stroke and (3) inability to undergo cognitive testing due to severe aphasia, as determined by the attending physician.

All subjects provided informed written consent in accordance with the Declaration of Helsinki. The local institutional review boards approved the study protocol and waived the required consent requirements based on the retrospective nature of this study and the minimal risk to participants. An identical participant sample was used in previously published research^4,5^.

**Neuroimaging data pre-processing**

Whole-brain MRI scans were typically acquired within the first week after the stroke event. Brain scanning included structural axial T1, T2-weighted spin echo, fluid-attenuated inversion recovery and DWI sequences (3.0 T, Achieva scanner, Philips Healthcare, Netherlands, image dimensions: 182 × 218 × 182; c.f., Neuroimaging protocols for details). Stroke lesions were manually segmented on DWI or, less frequently, FLAIR images by experienced, trained investigators (A.K.K. and G.A.) relying on in-house developed software based on MeVisLab (MeVis Medical Solutions AG, Bremen, Germany)^6^. Lesion segmentations were successively checked and potentially refined by two experienced raters (N.A.W. and J.M.B). Subsequently, images and corresponding lesion maps were linearly and non-linearly normalized to Montreal Neurological Institute (MNI-152) space employing the RegLSM image processing pipeline (public code:<http://lsm.isi.uu.nl/>)^7^. An experienced rater rigorously controlled the quality of normalization (N.A.W.). If there were any visual differences between the original and registered lesion maps during quality control, normalized lesion maps were manually corrected.

**Neuroimaging protocols**

(1) Seoul National University Bundang Hospital: The MRI protocols comprised diffusion-weighted imaging (DWI), axial T1- and T2weighted spin echo, fluid-attenuated inversion recovery imaging (FLAIR), gradient-echo imaging, and coronal T1-weighted spin echo imaging. FLAIR imaging was acquired using a fast-spin echo sequence with the acquisition parameters: repetition time: 11,000 ms; echo time: 125 ms; inversion time: 2800ms; slice thickness 5 mm; intersection gap 1mm; matrix: 512×512; flip angle 90 degrees. DWI imaging was obtained employing an EPI-spin echo sequence with the acquisition parameters: repetition time: 5000 ms; echo time: 50 ms; diffusion b-value: 1000; slice thickness: 5 mm; intersection gap: 1 mm; matrix: 256 × 256; flip angle 90 degrees. (2) Hallym University Sacred Heart Hospital: The MRI protocols comprised DWI, axial T1- and T2-weighted spin echo, FLAIR, gradient-echo imaging, and coronal T2-weighted spin echo imaging. FLAIR imaging acquisition parameters: repetition time: 11,000 ms; echo time: 125 ms; inversion time: 2800 ms; slice thickness: 5 mm; matrix: 512×512; flip angle 90 degrees. DWI image acquisition parameters: repetition time: 3000ms; echo time: 56ms; diffusion b-value: 1000; slice thickness: 5 mm; matrix: 256 ×256; flip angle 90 degrees.

**Latent factors driving cognitive outcomes**

Factor analysis re-represents the set of cognitive scores with a smaller set of hidden factors while maximizing the amount of explained correlation or, in other words, common variance (the amount of variance that is shared among cognitive scores). This common variance, along with unique variance (any portion of variance that is not shared among the 8 cognitive endpoints), makes up the total variance. In contrast, the commonly used principal components analysis assumes that there is no unique variance and the total variance is equal to the common variance.

The factor analysis model treats observed cognitive scores Y as measures of a smaller number of unobserved latent factors F, with corresponding loadings A, such that:

$$Y\approx FA'+E,$$

where Y is the [*n* × *l*] matrix of *l* centered (de-meaned across participants) and standardized (unit-variance scaled across participants) cognitive scores for *n* individuals, F is the [*n* × *p*] matrix of *n* individuals’ values for *p* latent variables, A is the [*l* × *p*] matrix of the latent variable effects on the *l* cognitive scores, and E represent score-specific [*n* x *l*] matrix of unique disturbance terms.

The weights A in the factor analysis express the relationship or association of each score Y to the underlying factor F. To estimate the parameters of the dimensionality reduction, the factor analysis finds a matrix of loadings A and a diagonal matrix Y such that the observed covariance matrix S is as well as possible approximated by:

$$\Sigma\approx AA'+\Psi$$

In other words, the factor analysis minimizes differences between off-diagonal elements of S and A. Since we can choose the diagonal matrix Y, the reconstruction error of the diagonal elements will be zero. While several methods exist for the extraction of latent factors, we selected the widely employed “varimax” solution that estimates factor loadings while minimizing the sum of squares of off-diagonal residuals^8^.

Due to rotational indeterminacy (an infinite number of equivalent A matrices up to a particular rotation), we chose the commonly used “varimax” rotation. This method minimizes the number of variables that have high loadings on each factor and thus simplifies the interpretation of the factors. Based on the inspection of explained variance and associated eigenvalues, we opted for a four-factor solution. This solution reaches a compromise between the detailed and data-efficient characterization of the overarching cognitive dimensions. The derived underlying driving factors among our 8 target cognitive scores served as the basis for our modelling outcomes in downstream analyses.

**Bayesian Model specification**

Our generative, multi-level approach allowed us to obtain fully probabilistic parameter estimates that could inform us about effect strength and effect certainty of how each lesion pattern is responsible for the respective outcome. To directly examine possible differences in hemispheric predictive relevance for the selected outcome, the modelled generative process assumed a joint dispersion prior for all lesion atoms of each hemisphere. Therefore, the standard deviation priors for the left and right hemispheres could capture the hemisphere-specific predictive contributions tiled across all candidate lesion atoms. Priors of left- and right-hemispheric standard deviations were additionally combined through a joint hyperprior to complement the hierarchical model structure.

For all analytical solutions, samples from the joint posterior distribution of the model parameters were drawn by the No U-Turn Sampler, a Monte Carlo Markov Chain algorithm (setting: draws = 4000)^9^. Posterior predictive checks were carried out after model estimation to evaluate the obtained predictive model (with respect to its R^2^-based explained variance). In other words, we empirically assessed the simulated outcome predictions generated by our model solution to approximate external validation based on our patient sample. This empirical procedure is a well-recognized option for judging the adequacy of Bayesian models given the actual data at hand^10,11^.

**Full Bayesian model specification for MIMO model**

**Hyperpriors**

$$\boldsymbol{hype}\boldsymbol{r}_{\boldsymbol{\sigma}_{\boldsymbol{\beta}}}\boldsymbol{\sim Halfcauchy(\beta=1)}$$

$$\sigma_{\beta}\sim\boldsymbol{hype}\boldsymbol{r}_{\boldsymbol{\sigma}_{\boldsymbol{\beta}}}$$

**Priors**

$$\alpha\sim\boldsymbol{Normal(\mu=0, \sigma=1)}$$

$\beta_{1-10}\sim\boldsymbol{Normal(\mu=0, \sigma=}\boldsymbol{\sigma}_{\boldsymbol{\beta}}\boldsymbol{)}$

$\beta_{lesion load}\sim\boldsymbol{Normal(\mu=0, \sigma=10)}$

$\beta_{age}\sim\boldsymbol{Normal(\mu=0, \sigma=10)}$

$\beta_{{age}^{2}}\sim\boldsymbol{Normal(\mu=0, \sigma=10)}$

$\beta_{male}\sim\boldsymbol{Normal(\mu=0, \sigma=1)}$

$\beta_{female}\sim\boldsymbol{Normal(\mu=0, \sigma=1)}$

$\beta_{education years}\sim\boldsymbol{Normal(\mu=0, \sigma=5)}$

$\beta_{IQCODE}\sim\boldsymbol{Normal(\mu=0, \sigma=1)}$

$\beta_{time since onset}\sim\boldsymbol{Normal(\mu=0, \sigma=10)}$

**Likelihood of individual linear models (example for MMSE)**

$Y_{MMSE}=\alpha+\beta_{1-10}[hemisphere]+\beta_{lesion load}*Lesion load+\beta_{age}*age+\beta_{age^{2}}*age^{2}+$

$\beta_{male}*male+\beta_{female}*female+\beta_{education years}*education years+$

$\beta_{IQCODE}*IQCODE+\beta_{time onset}*time onse$t


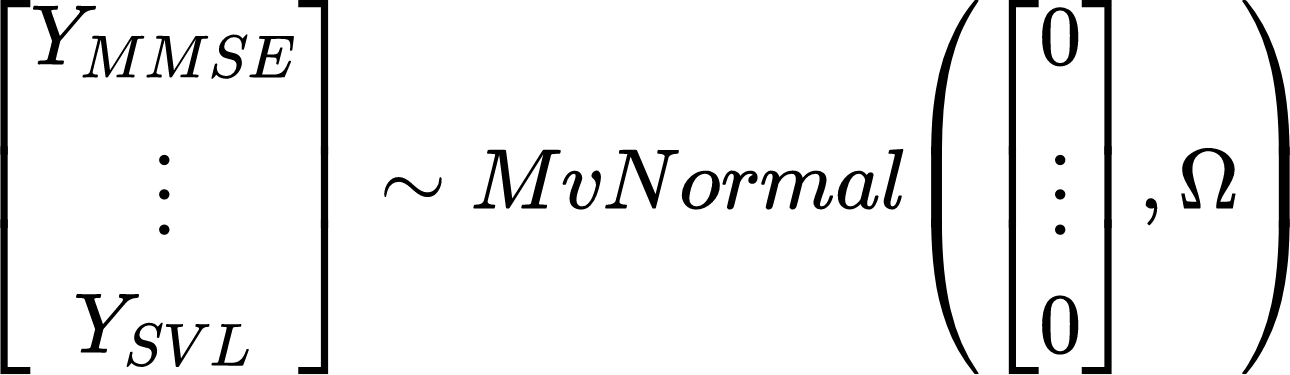

$$\Omega=LKJCorr(\eta=4, sd dist=Exponential(1))$$

**Full Bayesian model specification for MISO model (example for Factor 1)**

**Hyperpriors**

$$\boldsymbol{hype}\boldsymbol{r}_{\boldsymbol{\sigma}_{\boldsymbol{\beta}}}\boldsymbol{\sim Halfcauchy(\beta=1)}$$

$$\sigma_{\beta}\sim\boldsymbol{hype}\boldsymbol{r}_{\boldsymbol{\sigma}_{\boldsymbol{\beta}}}$$

**Priors**

$$\alpha\sim\boldsymbol{Normal(\mu=0, \sigma=1)}$$

$\beta_{1-10}\sim\boldsymbol{Normal(\mu=0, \sigma=}\boldsymbol{\sigma}_{\boldsymbol{\beta}}\boldsymbol{)}$

$\beta_{lesion load}\sim\boldsymbol{Normal(\mu=0, \sigma=10)}$

$\beta_{age}\sim\boldsymbol{Normal(\mu=0, \sigma=10)}$

$\beta_{{age}^{2}}\sim\boldsymbol{Normal(\mu=0, \sigma=10)}$

$\beta_{male}\sim\boldsymbol{Normal(\mu=0, \sigma=1)}$

$\beta_{female}\sim\boldsymbol{Normal(\mu=0, \sigma=1)}$

$\beta_{education years}\sim\boldsymbol{Normal(\mu=0, \sigma=5)}$

$\beta_{IQCODE}\sim\boldsymbol{Normal(\mu=0, \sigma=1)}$

$\beta_{time since onset}\sim\boldsymbol{Normal(\mu=0, \sigma=10)}$

**Likelihood of linear model**

$$Factor1=\alpha+\beta_{1-10}[hemisphere]+\beta_{lesion load}*Lesion load+\beta_{age}*age+\beta_{age^{2}}*age^{2}+$$

$\beta_{male}*male+\beta_{female}*female+\beta_{education years}*education years+$

$\beta_{IQCODE}*IQCODE+\beta_{time onset}*time onse$t

$$\varepsilon\sim Halfcauchy(\beta=20)$$

$$Factor1\sim Normal(\mu=Factor1,\sigma=\varepsilon)$$

**Results**

Additional results for the lesion atom analyses are summarized in Supplementary Figure 1.

***
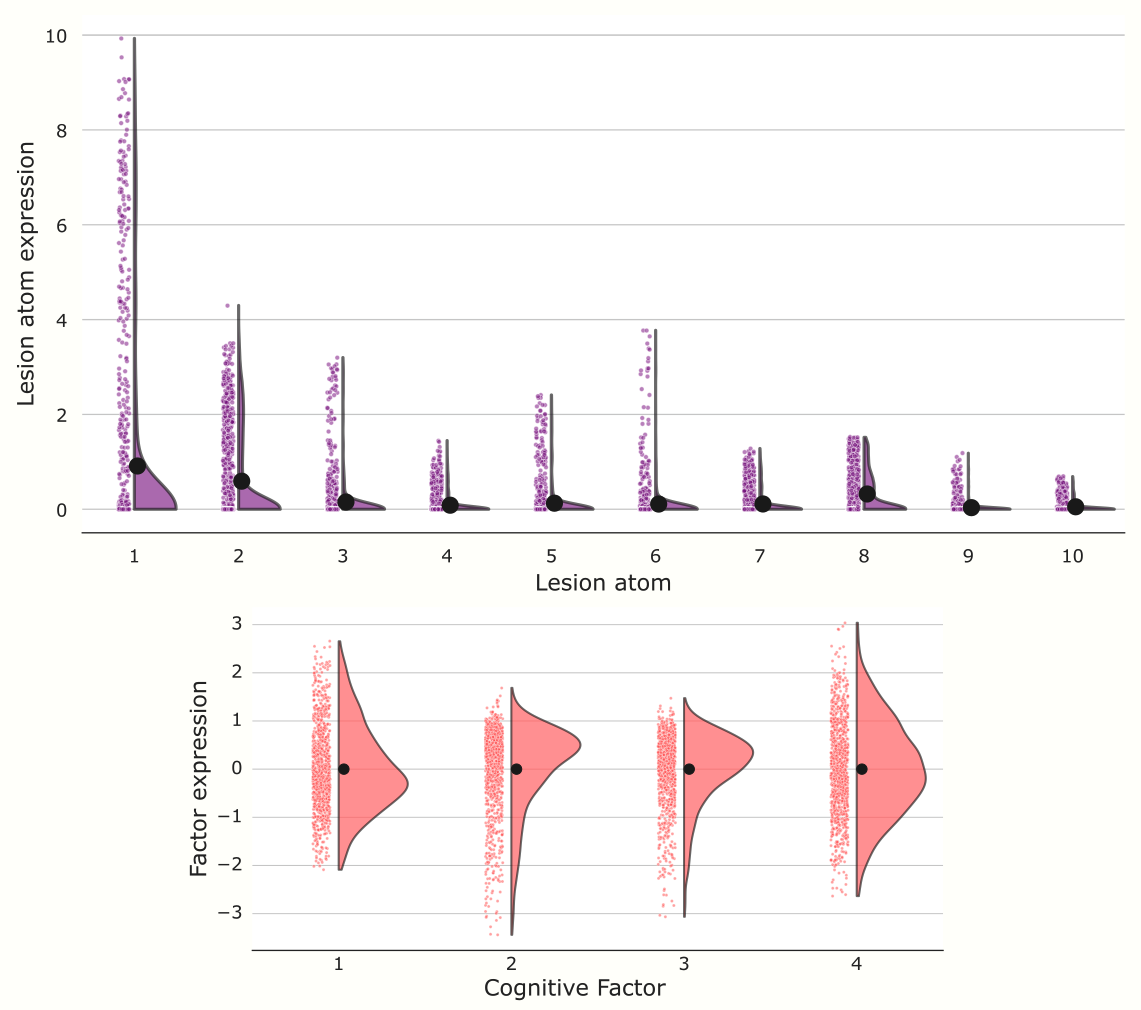
***

**Supplementary Figure 1. Subject scores across lesion atoms and cognitive factors.** The top row depicts the similarity between the derived 10 coherent topographical patterns and each patient’s lesion fingerprint. Therefore, stronger “lesion atom expression” indicates a stronger resemblance to the respective topographical lesion pattern. Similarly, the bottom row depicts the similarity between factor-analysis-derived overarching domains and the cognitive profile of each subject. Again, stronger “factor expression” indicates a stronger resemblance to the respective cognitive factor.

**Multiple-input multiple-output model posterior estimate distributions**

We designed a single multivariate multivariable Bayesian hierarchical model aimed to explain interindividual differences in eight cognitive outcome scores. In doing so, we obtained full posterior Bayesian estimates of the parameter distribution to infer the explanatory relevance of each lesion atom (NNMF factors 1-10) for the prediction of each of the eight cognitive scores in patients with stroke. The highest density intervals (HDI) of the posterior distribution showed based on 94% (default in PyMC3) certainty as depicted as a black horizontal line in the following plots.

**Multiple-input multiple-output model posterior estimate distributions**

We further designed four multivariate single-variable Bayesian hierarchical model aimed to explain interindividual differences in four factor scores. In doing so, we obtained full posterior Bayesian estimates of the parameter distribution to infer the explanatory relevance of each lesion atom (NNMF factors 1-10) for the prediction of each of the four factor scores in patients with stroke. The highest density intervals (HDI) of the posterior distribution showed based on 94% (default in PyMC3) certainty as depicted as a black horizontal line in the following plots.

**Supplementary References**

1. Kim BJ, Park JM, Kang K, et al. Case Characteristics, Hyperacute Treatment, and Outcome Information from the Clinical Research Center for Stroke-Fifth Division Registry in South Korea. *J Stroke*. 2015;17(1):38-53.

2. Yu KH, Cho SJ, Oh MS, et al. Cognitive impairment evaluated with Vascular Cognitive Impairment Harmonization Standards in a multicenter prospective stroke cohort in Korea. *Stroke*. 2013;44(3):786-788.

3. Hachinski V, Iadecola C, Petersen RC, et al. National Institute of Neurological Disorders and Stroke-Canadian Stroke Network vascular cognitive impairment harmonization standards. *Stroke*. 2006;37(9):2220-2241.

4. Bonkhoff AK, Lim JS, Bae HJ, et al. Generative lesion pattern decomposition of cognitive impairment after stroke. *Brain Communications*. 2021;3(2):fcab110.

5. Kernbach JM, Hartwigsen G, Lim JS, et al. Bayesian stroke modeling details sex biases in the white matter substrates of aphasia. *Commun Biol*. 2023;6(1):354.

6. Ritter F, Boskamp T, Homeyer A, et al. Medical image analysis. *IEEE Pulse*. 2011;2(6):60-70.

7. Weaver NA, Zhao L, Biesbroek JM, et al. The Meta VCI Map consortium for meta-analyses on strategic lesion locations for vascular cognitive impairment using lesion-symptom mapping: Design and multicenter pilot study. *Alzheimers Dement (Amst)*. 2019;11:310-326.

8. Harman HH, Jones WH. Factor analysis by minimizing residuals (minres). *Psychometrika*. 1966;31(3):351-368.

9. Hoffman, M.D., Gelman, A. The No-U-Turn sampler: adaptively setting path lengths in Hamiltonian Monte Carlo. *J Mach Learn Res*. (15):1593-1623.

10. Kruschke J. Doing Bayesian Data Analysis: A Tutorial with R, JAGS, and Stan. In: ; 2014. Accessed January 6, 2023.

11. Gelman A, Carlin JB, Stern HS, Dunson DB, Vehtari A, Rubin DB. *Bayesian Data Analysis*. 3rd ed. Chapman and Hall/CRC; 2015. doi:10.1201/b16018
